# Supplementary material for: Femtosecond electron beam probe of ultrafast electronics
Source: Nat Commun. 2024 Feb 26;15:1743. doi: 10.1038/s41467-024-45744-8 (PMC10897311; doi:10.1038/s41467-024-45744-8)
Supplement: Supplementary file 1 — Supplementary Information [file 41467_2024_45744_MOESM1_ESM.pdf]

Supplementary Information for

Femtosecond electron beam probe of ultrafast electronics

Maximilian Mattes, Mikhail Volkov and Peter Baum
Universität Konstanz, Universitätsstraße 10, 78464 Konstanz, Germany

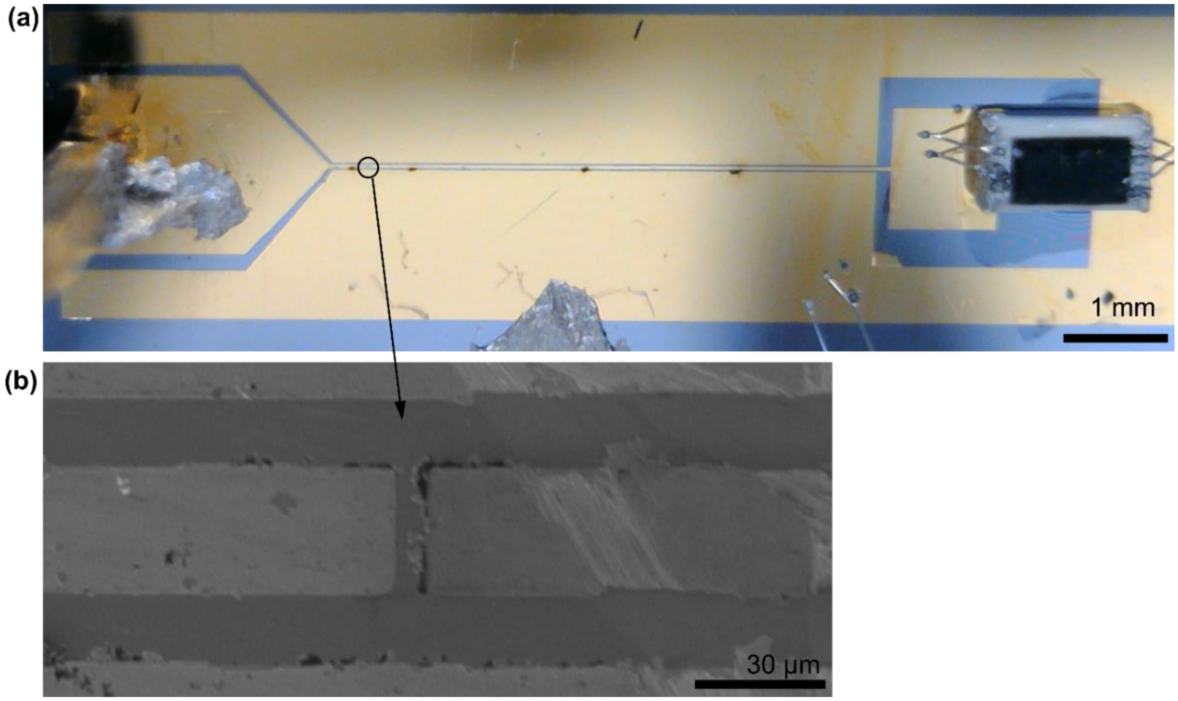

Supplementary Fig. 1. Microscope images of the chip. **(a)** Optical microscope image of the mounted chip with the bias connection on the left and the 50 Ohm resistor on the right. **(b)** Scanning electron microscope image of the photoconductive gap and the waveguide.

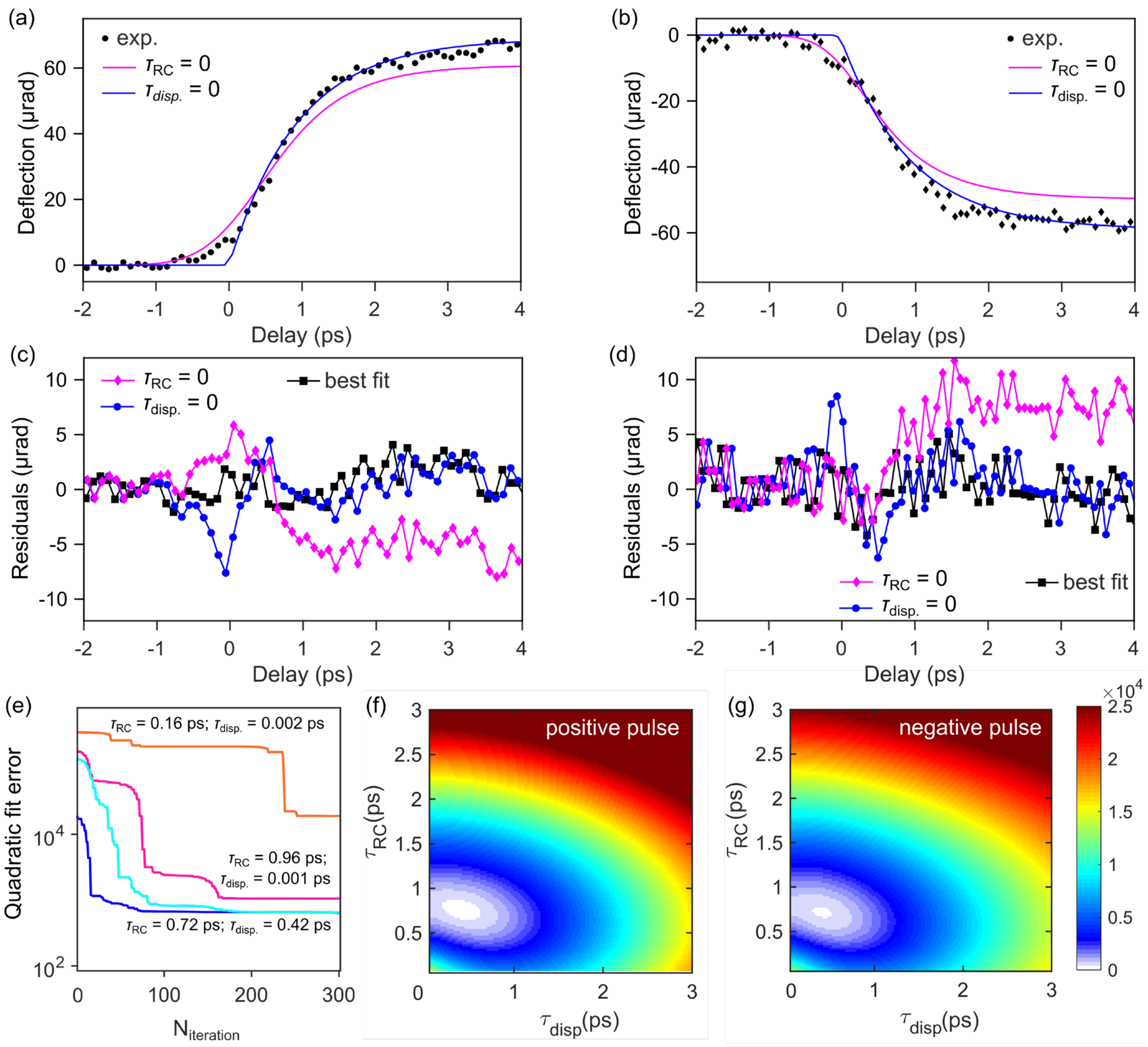

Supplementary Fig. 2. Various fits and their residuals. **(a), (b)** Fits of the experimental data (black dots and diamonds) with a model assuming RC-dynamics and the finite electron pulse duration but no voltage pulse dispersion (blue), and another model, assuming only dispersion but no RC-dynamics (magenta). **(c), (d)** Fit residuals corresponding to panels (a), (b). For comparison, the black squares show the residuals obtained with the model from eq. (1). (e) Residual fit error for different initial conditions. Orange and magenta, unphysical initial guesses with close-to-zero dispersive broadening time. Blue, cyan, realistic initial guesses. (f) Map of fitting errors for positive voltage pulses as a function of τ_{RC} and τ_{disp}. (g) Map of fitting errors for negative pulses. The smallest errors (white colour) show a single global minimum in both maps.
